# Supplementary material for: Surface Modification of Fumed Silica by Plasma Polymerization of Acetylene for PP/POE Blends Dielectric Nanocomposites
Source: Polymers (Basel). 2019 Nov 28;11(12):1957. doi: 10.3390/polym11121957 (PMC6960918; doi:10.3390/polym11121957)
Supplement: Supplementary file 1 [file polymers-11-01957-s001.pdf]

Surface Modification of Fumed Silica by Plasma Polymerization of Acetylene for PP/POE Blends Dielectric Nanocomposites

Xiaozhen He [1,*], Ilkka Rytöluoto [2], Rafal Anyszka [1,*], Amirhossein Mahtabani [1], Eetta Saarimäki [2], Kari Lahti [3], Mika Paajanen [2], Wilma Dierkes [1,*] and Anke Blume [1]

[1] Elastomer Technology and Engineering, Department of Mechanics of Solids, Surfaces & Systems (MS3), Faculty of Engineering Technology, University of Twente, P.O.box 217, 7500 AE, Enschede, The Netherlands; a.mahtabani@utwente.nl (A.M.); a.blume@utwente.nl (A.B.)

[2] VTT Technical Research Centre of Finland Ltd., FI-33101 Tampere, Finland; Ilkka.Rytoluoto@vtt.fi (I.R.); Eetta.Saarimaki@vtt.fi (E.S.); Mika.Paajanen@vtt.fi (M.P.)

[3] High Voltage Engineering, Tampere University, FI-33720 Tampere, Finland; kari.lahti@tuni.fi

[*] Correspondence: w.k.dierkes@utwente.nl (W.K.D.); r.p.anyszka@utwente.nl (R.P.A.); x.he@utwente.nl (X.H.)

Supplementary Material

S1. Measurement of isothermal polarization and thermally stimulated depolarization current

Isothermal polarization current and thermally stimulated depolarization current (TSDC) measurements were carried out in order to study the transient electrical conductivity and charge trapping behavior of the nanocomposite samples (see Fig. S1). There are three main regions during the measurement:

1. Isothermal polarization;
2. Cooling (while still maintaining the electric field);
3. Thermally stimulated depolarization (no field applied).

The charges were injected into the sample during the poling phase and then kept for stabilization during freezing. During the polarization step, the measured isothermal charging current data provided an indication of the transient electrical conductivity behavior of the sample. During the subsequent depolarization step, when the sample was heated linearly under short-circuit conditions, gradual relaxation of (dipolar) polarization and trapped charge release occurs, giving rise to a thermally stimulated depolarization current (TSDC) in the external circuit. In principle, for non-polar polymers, the TSDC above the glass transition temperature is mostly attributable to space charge relaxation, with the temperature at peak maximum and the peak intensity being related to the depth and density of the charge traps, respectively.

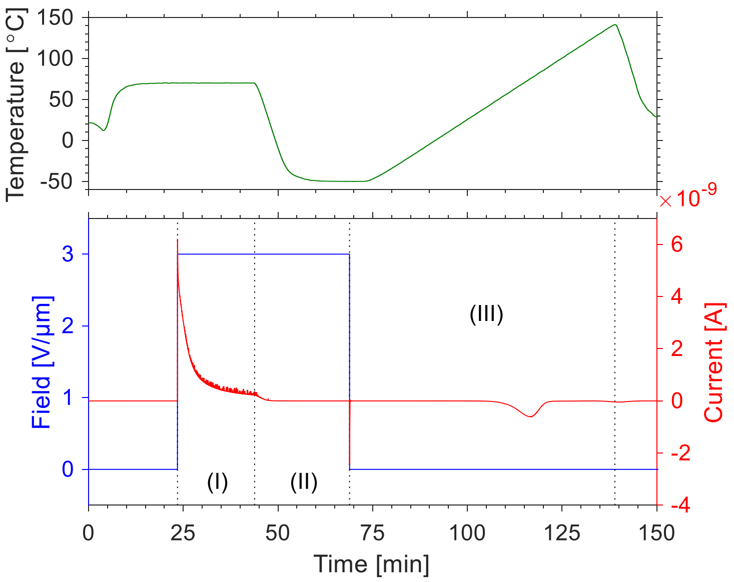

Figure S1. Exemplifying polarization/depolarization current of a plasma modified silica filled PP/POE composite. The annotated regions are (I): isothermal polarization, (II): cooling while still maintaining the electric field and (III): thermally stimulated depolarization.

S2. Determination of trap density distribution from TSDC data

Assuming that only electrons are injected into the sample during the high-field polarization, the induced thermally stimulated depolarization current density during linear heating can be expressed as [1,2]:

$$J(T) = \frac{ql^2}{2d}\int_{E_v}^{E_c} f_0(E)N_t(E)e_n(E,T)\exp\left(-\frac{1}{\beta}\int_{T_0}^{T} e_n(E,T)dT\right)dE \tag{1}$$

where

$$e_n(E,T) = v\exp(-E_t/kT) \tag{2}$$

In (1) and (2), q is the elementary charge, l is the penetration depth, d is the sample thickness, f_0 is the initial occupancy of electron traps, $N_t(E)$ is the trap level density distribution function, E is the trap depth, T is temperature, β is the heating rate, T_0 is the cooling temperature, $e_n(E,T)$ is the rate of emission of electrons, v is the attempt-to-escape frequency and k is the Bolzmann constant. For the calculations it was assumed that the all the traps were initially filled ($f_0 = 1$) and the penetration depth of the injected charge layer was l = 5 μm. The attempt-to-escape frequency was taken as $v = kT/h$ where h is Planck's constant. Thus, within the experimental temperature range (from −50 °C to 140 °C), the attempt-to-escape frequency varies from $v = 4.65 \times 10^{12}$ s^{-1} to $v = 8.61 \times 10^{12}$ s^{-1} which is within the physically acceptable range.

As originally proposed by Simmons [1], a new function $G(E,T)$, appearing in the integrand of (1), can be defined as:

$$G(E,T) = e_n(E,T)\exp\left(-\frac{1}{\beta}\int_{T_0}^{T} e_n(E,T)dT\right) \tag{3}$$

The form of $G(E,T)$ is an asymmetrical bell-shaped curve which, according to the analyses provided by both Simmons [1] and Tian et al. [2], can be approximated as a δ function:

$$G(E,T) = A(E_m)\delta(E - E_m) \tag{4}$$

where E_m corresponds to the trap level at peak maximum of $G(E,T)$ at temperature T. $A(E_m)$ at each temperature T can be solved by numerical method provided by Tian *et al.* [2]. Finally, the trap density distribution $N_t(E)$ can be solved as:

$$N_t(E_m) = \frac{2dJ(T)}{ql^2A(E_m)f_0} \tag{5}$$

[1] J.G. Simmons, G.W. Taylor, M.C. Tam, Thermally Stimulated Currents in Semiconductors and Insulators Having Arbitrary Trap Distributions, Phys. Rev. B. 7 (1973) 3714–3719. doi:10.1103/PhysRevB.7.3714.

[2] F. Tian, W. Bu, L. Shi, C. Yang, Y. Wang, Q. Lei, Theory of modified thermally stimulated current and direct determination of trap level distribution, J. Electrostat. 69 (2011) 7–10. doi:10.1016/j.elstat.2010.10.001.
